# Multiple social factors are associated with wellbeing when accounting for shared genetic and environmental confounding

**DOI:** 10.1007/s11136-024-03832-8

**Published:** 2024-11-20

**Authors:** Ludvig Daae Bjørndal, Ragnhild Bang Nes, Ziada Ayorech, Olav Vassend, Espen Røysamb

**Affiliations:** 1 PROMENTA Research Center, Department of Psychology, University of Oslo, Oslo, Norway; 2 Division of Mental and Physical Health, Norwegian Institute of Public Health, Oslo, Norway; 3 Department of Philosophy, Classics, and History of Arts and Ideas, University of Oslo, Oslo, Norway; 4 Department of Psychology, University of Oslo, Oslo, Norway

**Keywords:** Social factors, Wellbeing, Genetics, Environmental influences, Twin study, Co-twin control

## Abstract

**Purpose:**

Social factors are associated with mental health and wellbeing. However, few studies have examined genetic and environmental influences on social factors themselves, limiting current understanding of influences on aspects of the social environment. Most studies which have identified links between social factors and mental health are also limited by the possible influence of unmeasured genetic and environmental confounding. In this study, we investigated the genetic and environmental underpinnings of multiple social factors (relationship satisfaction, loneliness, attachment, trust, relationship disruptions), and their associations with life satisfaction measured concurrently and six years later, after accounting for shared genetic and environmental confounding.

**Methods:**

Data from a population-based sample of adult twins (N = 1987) and two measurement timepoints were used for the primary analyses. We used multivariate Cholesky models to estimate genetic and environmental influences across five social factors. Subsequently, we conducted co-twin control analyses to examine associations between social factors and wellbeing after controlling for shared genetic and environmental confounding.

**Results:**

Heritability estimates for the social factors ranged from 24 to 42%. Genetic correlations across social factors were substantial, indicative of considerable genetic overlap. Associations between wellbeing and relationship satisfaction, loneliness, anxious and avoidant attachment, trust, and disruptions in relationships in the past year were attenuated in co-twin control analyses but remained statistically significant. Relationship satisfaction, loneliness, and attachment avoidance were also associated with wellbeing measured six years later in estimates which controlled for shared genetic and environmental confounding.

**Conclusion:**

Our findings provide evidence that multiple social factors are associated with wellbeing after accounting for potential confounding by shared genetic and/or environmental factors. These findings highlight the importance of multiple aspects of the social environment for wellbeing in older adulthood. Future studies should examine the directionality in associations between social factors and mental health and assess these relationships beyond older adulthood.

**Supplementary Information:**

The online version contains supplementary material available at 10.1007/s11136-024-03832-8.

## Introduction

Humans are profoundly social beings and social relationships represent key influences on physical and mental health. Numerous aspects of the social environment influence health in both humans and several other species [60]. Social disconnectedness has been hailed as a major risk factor with detrimental health effects comparable to that of smoking [44], and loneliness and isolation is associated with strongly increased risk of early mortality [28]. Consequently, social adversity has recently been highlighted as an important public health concern [26].

Social factors also influence the risk of mental disorders. Disruptions in social relationships, such as divorce and conflict, constitute stressful life events associated with increased depression risk [34]. Conversely, experiencing social support was found to reduce the risk of depression by 55% in a large U.S. sample during the COVID-19 pandemic [12], whereas dissatisfaction with the partner relationship has been associated with emotional distress [54]. Variables such as social integration, social support, and early-life adversity are incorporated in aetiological models of mental disorders (e.g., the biopsychosocial model; [21]), underlining the critical importance of social factors for mental health.

The importance of social factors for wellbeing is emphasised in influential theories. For instance, multiple theories concerning wellbeing components (i.e., theories which aim to explain what wellbeing ‘consists of’), incorporate social factors as an integral aspect of the wellbeing construct itself [35, 57, 58]. Self-determination theory also conceptualises experiencing relatedness to others as a fundamental need required for experiencing good mental health: ‘an organismic necessity’ [56], p. 295). Baumeister & Leary [7] proposed the ‘belongingness hypothesis’: people seek and wish for lasting bonds to other people. Thus, these different proposed theories all point to various social factors as critical for good mental health.

A number of empirical studies have supported that multiple aspects of social relationships are related to wellbeing. Beneficial social relationships and interactions are similarly crucial for people’s wellbeing and thriving [27]. For instance, experiencing the social environment as positive and supportive is strongly linked with higher wellbeing in the general population [8], and being happy in one’s romantic relationship is associated with higher life satisfaction [19]. Social trust has been associated with wellbeing following natural disasters [66]. Several studies have also found that attachment styles and orientations are linked to wellbeing (e.g., [38, 43]). In contrast, loneliness has been associated with lower wellbeing with moderate effect size estimates across studies [51]. However, few studies have provided a thorough examination of how multiple social factors are related to wellbeing. This may inform theories concerning how such constructs are related to wellbeing and the relative importance of various social factors for mental health.

Individual differences in wellbeing are moderately influenced by genetic effects with the majority of differences explained by environmental variation [3, 47]. Several social factors also show genetic influence, such as loneliness [24], social support [31], attachment style [10, 52], and disruptions in social relationships (e.g., divorce; [30, 42]). While genetic effects across different wellbeing constructs are largely overlapping (e.g., [4, 9]), few studies have investigated the genetic and environmental underpinnings of social variables which may influence wellbeing in multivariate analyses. Such analyses can yield insights into the overlap of genetic and environmental effects on social factors, yielding a more comprehensive understanding of how genetic and environmental factors broadly relate to different social variables.

Genetic effects on social factors aligns with research showing that many measures of supposedly ‘pure’ environmental factors reflect substantial genetic influence [32]. Most studies of social factors and wellbeing do not account for possible genetic confounding. For instance, an observed association between relationship satisfaction and wellbeing could reflect genetic factors predisposing individuals to experience both higher wellbeing and higher satisfaction with their relationships (e.g., positivity orientation). Alternatively, observed associations could be confounded by influences in the early rearing environment shared between siblings. Promoting wellbeing is a UN Sustainable Development Goal [61] and an aim for societies worldwide. Thus, bettering the understanding of how social factors are linked to wellbeing, including the extent to which observed associations could reflect unmeasured confounding, is of critical importance for public health efforts striving to improve wellbeing in the population.

The co-twin control design is a powerful design which leverages the relatedness between twins to increase control over potential genetic and shared environmental confounding [41, 64]. The design compares outcomes for twins who are discordant in exposure to a risk factor, such as comparing wellbeing among twin pairs in which one twin experiences loneliness and the other does not. As genes are fully shared by monozygotic (MZ) twins, who also share the early rearing environment, this increases control over confounding by these familial factors.

Few studies have applied the co-twin control design to study social factors and wellbeing. One study found that bereavement is associated with reduced life satisfaction after accounting for shared genetic and environmental factors [37]. A recent twin study identified genetic influence on associations between social factors (e.g., friendship satisfaction) and wellbeing in adolescents [62]. Several studies have applied the co-twin control design and found that negative interpersonal experiences (e.g., victimisation) adversely affect mental health [2, 14, 33, 59]. Applying sibling designs to examine which social factors are related to wellbeing when adjusting for familial confounding could have implications for theories which relate these, given the possibility of confounding bias in previous (non-genetically informed) research.

In this study, we applied a two-fold approach to advance the current understanding of genetic and environmental influences on social factors and their associations with wellbeing in a population-based sample of Norwegian adult twins. ‘Wellbeing’ was conceptualised as life satisfaction, which is a key component in the subjective wellbeing (SWB) model [15, 17, 18]. Our analysis strategy involved first estimating genetic and environmental effects on six social factors—relationship satisfaction, disruptions in relationships, attachment anxiety, attachment avoidance, loneliness, and trust—and the extent to which these effects were overlapping or unique to each social factor. Following this, we conducted co-twin control analyses to examine associations between social factors and wellbeing while accounting for unmeasured confounding from shared genetic and environmental factors. Associations were examined both for wellbeing measured at the same timepoint (i.e., concurrently) and six years later, to examine if patterns of associations were consistent when wellbeing was measured at different timepoints.

## Methods

The present study was approved by the Regional Committees for Medical and Health Research Ethics (project numbers: 2015/958 and 2021/27872).

## Sample

We used two waves of data collection from a cohort of the Norwegian Twin Registry (NTR) born between 1945 and 1960. The first wave of data collection was conducted in 2016 [48]. The response rate was 64%. Data were collected from 1987 individuals in total. Of these, 528 were monozygotic (MZ) female twins, 627 dizygotic (DZ) female twins, 375 MZ male twins, and 457 DZ male twins. The mean age was 63 years (SD = 4.5).

The second wave of data collection was conducted in 2022. The response rate was 35%. We used data collected in 2022 from individuals who also participated in the 2016 wave of data collection. Of these, 335 were MZ female twins, 371 DZ female twins, 236 MZ male twins, and 286 DZ male twins. The mean age was 68.5 years (SD = 4.4).

## Measures

### Wellbeing measures

Wellbeing was measured using the Satisfaction with Life Scale (SWLS; [16]). The SWLS consists of 5 items assessing agreement that life is close to ideal, life conditions are very good, satisfaction with life, feeling one has gotten the most important things in life, and that one would have changed little about life if given the chance to live again. The SWLS has been previously validated in Norwegian samples [13]. The response format was based on a 7-point scale ranging from 1 (‘Strongly disagree’) to 7 (‘Strongly agree’). Cronbach’s alpha for the SWLS at wave 1 of data collection (2016) was 0.91.

### Measures of social relationships

Relationship satisfaction was measured using items from the Relationship Satisfaction Scale (RSS; [55]). RSS comprises five items which ask about happiness with the partner relationship, problems in the relationship, perceiving the partner as understanding, satisfaction with the partner relationship, and agreement on how children should be raised. Response options were on a 6-point scale ranging from “Strongly disagree” (1) to “Strongly agree” (6). Cronbach’s alpha for RSS was 0.87.

The assessment of disruptions in social relationships was based on reporting of three possible stressful interpersonal experiences: divorce, separation or termination of cohabitation; large conflicts in the partner relationship; and problems or large conflicts with family, friends or neighbours. Participants could report having experienced one or more such event in the last year or previously. A composite variable was created for which a score of 1 reflected having experienced any of these events and 0 not having experienced any events. For the co-twin control analyses, we assessed associations between disruptions in the past year and previously separately.

Attachment was measured using a short-form of the Experiences in Close Relationship Scale (ECR-N12) consisting of 12 items in total [50]. Response options were on a 7-point Likert scale ranging from “Strongly disagree” (1) to “Strongly agree” (7). ECR-N12 yields two scores, one which measures attachment anxiety and one which measures attachment avoidance. Cronbach’s alpha for the attachment anxiety subscale was 0.76 and for the attachment avoidance subscale was 0.84.

Loneliness was measured using a three-item version of the UCLA Loneliness scale [29]. Participants were asked if they feel isolated from others, lack someone to be with, and/or feel left outside. Response options were on a 5-point scale ranging from “Never” (1) to “Always” (5). Cronbach’s alpha for the UCLA loneliness scale was 0.87.

Trust was measured using three items adapted from the European Social Survey [49]. Participants were asked if they believe most people are to be trusted, that most people would try to take advantage of them if given the opportunity, and that most people try to be helpful. Response options were on a 10-point scale ranging from 0 to 10. Cronbach’s alpha for the Trust scale was 0.82.

### Loss to follow-up

Characteristics were broadly similar across participants with available SWLS data at both waves of data collection and one wave of data collection only. For the former (*N* = 1174), 57% were female and the mean age (during the 2016 wave of data collection) was 62.5 (*SD* = 4.38). For the latter (*N* = 741), 60% were female and the mean age was 63.9 (*SD* = 4.49). The mean SWLS sum score among participants with available SWLS data at both waves of data collection was 27.7 and one wave of data collection was 25.8, a difference which was statistically significant (*p* < 0.001).

## Statistical analyses

### Estimating genetic and environmental influences on aspects of social relations

We estimated genetic and environmental effects on the following social factors using multivariate Cholesky models: relationship satisfaction, attachment anxiety, attachment avoidance, loneliness, disruptions, and trust. The Cholesky model allows for the decomposition of covariance across traits into contributions from additive genetic effects (A), shared environmental effects (C), and non-shared environmental effects (E) [46]. Shared environmental effects contribute to increase similarity between twins, whereas non-shared environmental effects contribute to dissimilarity between twins (which may include systematic and random measurement error). The model also allows for estimating genetic and environmental correlations, i.e., the extent to which genetic and environmental influences on multiple traits are overlapping.

We compared the model fit of Cholesky models including ACE, AE, and E effects only. Model fit was assessed based on the Akaike Information Criterion (AIC [1];), with lower AIC values indicating better model fit. The data were residualised on age and sex prior to conducting these analyses. The biometric analyses were conducted using the *umx* (T. C. [6]) and *OpenMx* packages [45] in the R Statistical Environment [53].

### Examining associations between aspects of social relations and wellbeing controlling for shared environmental and genetic factors

The co-twin control design compares associations between MZ and DZ twins, who are correlated for genetic effects (MZ twins 1.0; DZ twins 0.5) and shared environmental effects (1.0 for both MZ and DZ twins), and uncorrelated for non-shared environmental effects [41, 64]. If associations are attenuated within MZ twin pairs compared with the full sample (i.e., all twins analysed together), this is indicative of partial confounding by either genetic or shared environmental factors. If effects remain robust within discordant twin pairs, this indicates that associations are not explained by unmeasured shared genetic or environmental confounding. Thus, the regression coefficient for the within-pair association reflects if a twin exposed to a social factor has higher or lower wellbeing over and above familial influences shared between the twins. The regression model can be represented as follows, where x_ij_ (in this example) denotes the level of a social factor (e.g., relationship satisfaction) for twin *j* in twin pair *i* and x_j_ the mean level of a social factor within twin pair *i* [11]:

$$y_{ij} = {\beta}_0 + {\beta}_{w} \left( {x_{ij} - \bar{x}_{i}} \right) + {\beta}_{B} \bar{x}_{i} + {\varepsilon}_{ij}$$

**Fig. 1:**
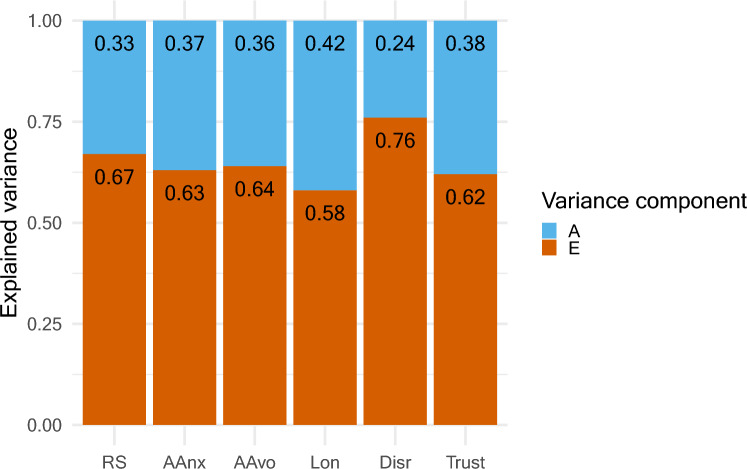
*Genetic and Environmental Effects on Social Factors*. These estimates were based on the multivariate AE Cholesky model. ‘RS’ represents relationship satisfaction; ‘AAnx’ represents attachment anxiety; ‘AAvo’ represents attachment avoidance; ‘Lon’ represents loneliness; and ‘Disr’ represents disrupted social relationships. ‘A’ refers to additive genetic effects. ‘E’ refers to non-shared environmental effects

We conducted co-twin control analyses with multilevel models using the *lme4* (D. [5]) and *lmerTest* [36] packages. The model examining associations in the full sample included a random intercept to account for dependence within each twin pair and the fixed effects of each social factor, sex, and age. The model for co-twin control analyses within MZ twins included a random intercept for the twin pairs and the fixed effects of the twin pair mean score (between-effect), the individual score subtracted from the mean score of the twin pair (within-effect), sex, and age. The outcome variable and within- and between-effects were standardized for all social factors except for disruptions in social relationships. Associations are displayed using forest plots from the *forestplot* package [25]. Effect sizes were interpreted following recently proposed recommendations, i.e., *r* = 0.05 indicative of a very small effect, *r* = 0.10 a small effect, *r* = 0.20 a medium effect, and *r* = 0.30 a large effect [23]. We examined associations between social factors and wellbeing measured concurrently and six years later. Complete case analysis was conducted so participants were required to have responded to all items to be included in analyses. The co-twin control analyses only used data from complete pairs, i.e., where both twins had responded to all items (incomplete pairs were included in full sample analyses). Sample sizes for the models examining cross-sectional associations ranged from 1509 to 1899 for each model conducted in the full sample and 438 to 656 for each model conducted in MZ twins. Sample sizes for models examining associations with SWLS measured six years later ranged from 978 to 1191 for each model conducted in the full sample and 244 to 356 for each model conducted in MZ twins. We report how we determined our sample size, all data exclusions, and all measures in the study. The sample size for each model is reported in the Supplementary Materials (Fig. 1).

## Results

### Genetic and environmental influences on social relations

The correlations between MZ twins were higher than for DZ twins for all social factors, which is indicative of genetic influence (see Supplementary Tables S1 – S4 for descriptive statistics, phenotypic correlations, and correlations in twin pairs). The AE Cholesky model had best fit to the data ($AIC=27779.04$). The heritability estimates ranged from 0.24 to 0.42 (see Fig. 1). The standardized parameter estimates from the AE Cholesky model are reported in the Supplementary (Tables S6 and S7), as are the fit statistics for all Cholesky models Table (S5). Thus, the best-fitting model did not include shared environmental effects.

**Fig. 2:**
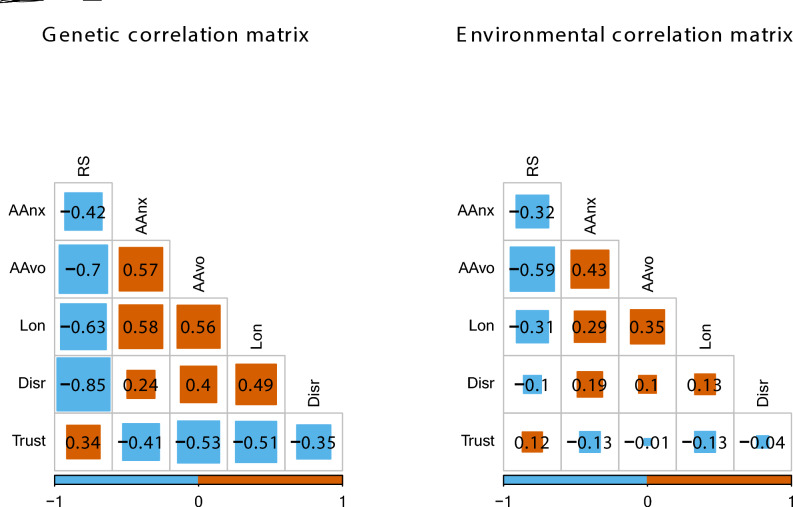
*Genetic and Environmental Correlations From AE Cholesky Model*. ‘RS’ represents relationship satisfaction; ‘AAnx’ represents attachment anxiety; ‘AAvo’ represents attachment avoidance; ‘Lon’ represents loneliness; and ‘Disr’ represents disrupted social relationships

**Fig. 3:**
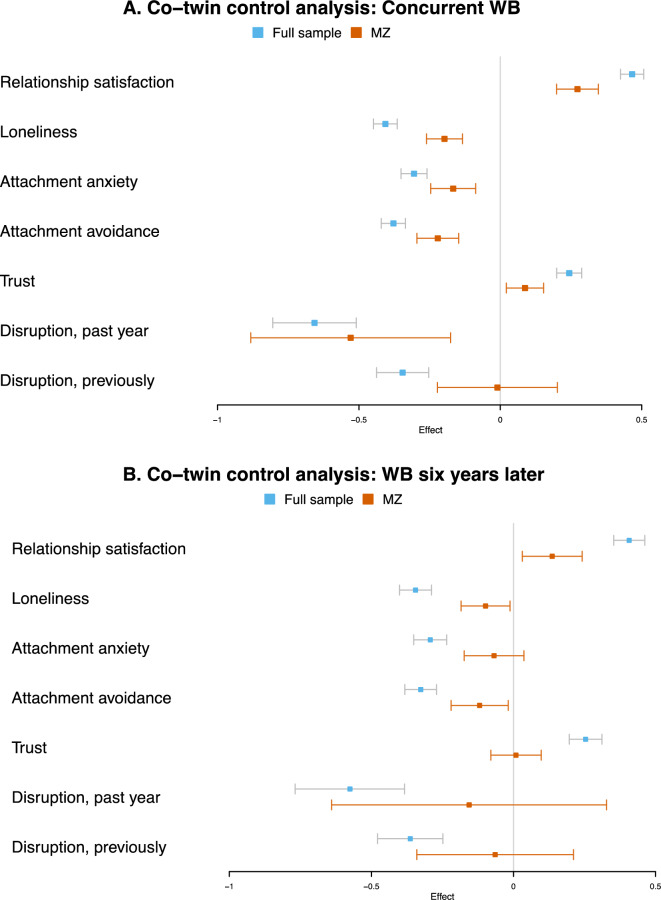
*Co-twin Control Analyses Examining Associations Between Social Factors and Wellbeing Measured Concurrently (A) and Six Years Later (B)*. ‘WB’ represents wellbeing. ‘MZ’ refers to within-pair estimates for monozygotic twins. The outcome and within- and between-effects were standardized for all social variables except for disruptions in social relationships. The error bars reflect 95% confidence intervals for the estimated effects

Genetic correlations across the social factors were substantial and ranged from 0.24 (between attachment anxiety and disruptions) to 0.85 (between relationship satisfaction and disruptions) in absolute values (see Fig. 2). Environmental correlations across the social factors were generally lower and ranged from 0.01 (between attachment avoidance and trust) to 0.59 (between relationship satisfaction and attachment avoidance) in absolute values. All correlations are reported in the Supplementary (Tables S7 and S8). (Fig. 3)

### Associations between aspects of social relations and wellbeing controlling for shared environmental and genetic factors

All social factors were associated with wellbeing measured concurrently in full sample analyses. Within-pair associations were attenuated but remained statistically significant for relationship satisfaction, loneliness, attachment anxiety, attachment avoidance, trust, and disruptions in relationships in the past year. The effect sizes for these within-pair effects ranged from small ($\beta = .09;\text{trust})$ to large ($\beta = -.53;\text{disruption in the past year})$. The within-pair effect was close to half of the full sample association for relationship satisfaction, loneliness, attachment anxiety and attachment avoidance. For instance, the within-pair effect $\beta$ for loneliness was, $- .20 \left( {95\% CI: - .26, - .13} \right)$ and the full sample association $\beta$ was$- .41 \left( {95\% CI: - .45, - .36} \right)$ All social factors were also associated with wellbeing measured six years later in full sample analyses. Stronger attenuation was observed in within-pair estimates compared with the cross-sectional associations but remained statistically significant for relationship satisfaction, loneliness, and attachment avoidance. The effect sizes for the within-pair effects were small, with the strongest effect size estimated for relationship satisfaction $(\beta = .14, 95\% CI:.03, .24)$. Attachment anxiety, trust, and disruptions in social relationships were not associated with wellbeing measured six years later in within-pair estimates. All fixed and random effects are reported in the Supplementary Materials.

## Discussion

We found that genetic influences were largely shared across different social factors while the overlap of environmental effects was lower. Diverse social factors were associated with wellbeing measured concurrently after accounting for genetic and environmental confounders shared between twins. Within-pair estimates were attenuated but associations remained substantial, indicative of partial confounding. Several social factors were also related to wellbeing measured six years later in analyses. These findings extend previous research on relationships between social factors and wellbeing in older adulthood.

Several social variables examined in our study have previously been associated with wellbeing, such as loneliness and relationship satisfaction [19, 51]. For instance, Park et al. [51] estimated a large effect size for the association between loneliness and wellbeing across 30 studies. In the present study, the within-pair association between loneliness and wellbeing which controlled for confounding was almost half of the full sample association but remained substantial. Taken together, our findings indicate that associations between diverse social factors and wellbeing are only partly explained by shared genetic and environmental confounding. This broadly underscores the importance of social factors for mental health and wellbeing in older adulthood. Nevertheless, this also suggests that associations between social factors and wellbeing in non-genetically informed studies can be biased by unmeasured confounding.

Disruptions in social relationships were strongly associated with lower wellbeing measured concurrently. This finding is broadly in agreement with previous genetically informed studies which have examined the effects of adverse interpersonal experiences on mental health [2, 33, 59]. This finding is also in agreement with one previous co-twin control study which found a negative effect of bereavement on life satisfaction within twin pairs [37]. We expand upon previous investigations by specifically examining the relationship between wellbeing and several interpersonal stressful life events (i.e., divorce or separation, conflicts with the partner, and conflicts with other people) in adults.

Three social factors (relationship satisfaction, loneliness, and attachment avoidance) were also associated with wellbeing measured six years later in estimates which controlled for shared genetic and environmental confounding. Previous studies have found that loneliness and wellbeing are associated across time and that effects may also be reciprocal [63]. A useful aim for future studies would be to examine if associations between social factors and wellbeing are robust to unmeasured confounding using longitudinal genetically informed designs.

To our awareness, no previous genetically informed study has examined the genetic and environmental underpinnings of multiple social factors in multivariate analyses. Previous studies have found evidence of genetic influence on associations between social factors and wellbeing in adolescents [62], and genetic correlations between social factors, such as loneliness and isolation, and mental disorders like depression, are high [40]. Previous studies have also found that influences on various components of wellbeing, including social aspects, are highly overlapping [9]. We here elaborated on these earlier findings by identifying substantial overlap in genetic effects across social factors.

Our study has several strengths and limitations. First, all measures of social factors have been validated and/or been used in previous research. Second, we used data from a population-based sample of twins recruited from the Norwegian Twin Registry. Third, by using data from two timepoints, we examined associations between social factors and wellbeing measured both concurrently and six years later. Nevertheless, our findings should be interpreted taking several limitations into account. Importantly, while the co-twin control design increases control over confounders shared between twins, it suffers from the potential influence of non-shared environmental confounding and measurement error [22]. We are also unable to shed light on directionality in observed associations. For instance, an association between relationship satisfaction and wellbeing measured at the same timepoint could reflect the influence of relationship satisfaction on wellbeing but also vice versa. A useful aim for future studies could be to use longitudinal data from genetically informed samples to disentangle the direction of effects. We only examined associations between social factors and wellbeing conceptualised as life satisfaction, which is a key component in the SWB model. However, future studies could use broader wellbeing measures. The mean age of our sample at the first wave of data collection was 63 years. The extent to which our findings generalise to other age groups is unclear, i.e., relationships between social factors and wellbeing in other age groups may not be similar to what was found for our sample. This is important also in light of studies which have found that some social difficulties, such as loneliness, may increase with age in adulthood (e.g., [65]). Finally, there was substantial loss to follow-up in our sample.

Our study has several potential implications for future research efforts. First, our results suggest that the heritability of several social phenotypes is substantial. This converges with studies which have identified small to moderate genetic effects across many environmental measures [32]. These findings highlight the importance of accounting for potential genetic confounding when examining associations between social factors and mental health in future studies, as such relationships from purely observational studies may in part reflect shared genetic and environmental confounding. Second, high genetic correlation across social factors suggests that genome-wide association studies examining genetic influences on social factors will find that many of the same genetic variants operate across different social phenotypes. Third, social difficulties represent an important risk factor for both adverse physical and mental health outcomes [26, 44]. While we find evidence of some confounding of observed associations between social factors and wellbeing by shared genetic and environmental factors, these relationships remain substantial also within twin pairs, i.e., they are not fully explained by unmeasured confounding.

Our findings may also have possible implications for clinicians and public health and prevention work. Our findings support the critical role of social factors for wellbeing in older adulthood. The importance of social factors for wellbeing is critical in light of recent studies reporting a small but robust increase in loneliness since the start of the COVID-19 pandemic [20]. Furthermore, our broad findings highlighting the importance of social factors for wellbeing in adulthood is highly relevant in the context of interventions which seek to lessen social difficulties such as loneliness [39]. Although future research is needed to determine directionality in associations, our findings indicate that the diverse social factors are robustly associated with wellbeing and could potentially represent useful targets for public health efforts seeking to increase wellbeing in the population. Finally, our findings can be taken to support the notion that social factors are critical for wellbeing and a good life, as has been emphasised also theoretically (e.g., [7, 56]).

## Conclusion

We examined the genetic and environmental architecture of social factors and associations between social factors and wellbeing in a population-based sample of adult twins. Heritability estimates ranged from 24 to 42% and genetic correlations were substantial across social factors. Multiple social factors were associated with concurrent and later wellbeing in within-pair estimates. Our results provide evidence that multiple social factors are associated with wellbeing after accounting for potential confounding by shared genetic and/or environmental factors, highlighting the importance of social factors for wellbeing in older adulthood. Nevertheless, associations between social factors and wellbeing in observational studies which do not account for confounding shared between twins or siblings may in part reflect bias from these factors.

## Supplementary Information

Below is the link to the electronic supplementary material.

Supplementary file1 (DOCX 79 KB)

## Data Availability

The R code used for all analyses is available in a public repository on the Open Science Framework: https://osf.io/u69dy/. The data from the NTR is not publicly available but may be requested from the registry itself (for more information, see https://www.fhi.no/en/more/health-studies/norwegian-twin-registry/). The study was not preregistered.
