# Correction: Multiple social factors are associated with wellbeing when accounting for shared genetic and environmental confounding

**DOI:** 10.1007/s11136-024-03886-8

**Published:** 2025-01-15

**Authors:** Ludvig Daae Bjørndal, Ragnhild Bang Nes, Ziada Ayorech, Olav Vassend, Espen Røysamb

**Affiliations:** 1 PROMENTA Research Center, Department of Psychology, University of Oslo, Oslo, Norway; 2 Division of Mental and Physical Health, Norwegian Institute of Public Health, Oslo, Norway; 3 Department of Philosophy, Classics, and History of Arts and Ideas, University of Oslo, Oslo, Norway; 4 Department of Psychology, University of Oslo, Oslo, Norway

**Correction: Quality of Life Research**

10.1007/s11136-024-03832-8

In the original published article, the incorrect equation below appearing in the subsection “**Examining associations between aspects of social relations and wellbeing controlling for shared environmental and genetic factors**”

$$yij\, = \,\beta 0\, + \,\beta w\,(xij\, - \,xi)\, + \,\beta Bxi\, + \,\varepsilon ij$$

should have been

$${y_{ij}}\, = \,{\beta _0}\, + \,{\beta _w}\,({x_{ij}}\, - \,\bar x{}_i)\, + \,\beta _B^{}{\bar x_i}\, + \,{\varepsilon _{ij}}$$

The original article has been corrected.

