# Supplementary material for: Multiple social factors are associated with wellbeing when accounting for shared genetic and environmental confounding
Source: Qual Life Res. 2024 Nov 20;34(2):535–45. doi: 10.1007/s11136-024-03832-8 (PMC11865172; doi:10.1007/s11136-024-03832-8)
Supplement: Supplementary file 1 — Supplementary file1 (DOCX 79 KB) [file 11136_2024_3832_MOESM1_ESM.docx]

**Table S1.**

*Descriptive Statistics for Key Measures.*

| **Wellbeing or social factor** | **Mean (SD)** | **Range** |
| --- | --- | --- |
| Wellbeing (mean score) | 5.39 (1.16) | 1 - 7 |
| Relationship satisfaction | 24.64 (4.48) | 5 - 30 |
| Attachment anxiety | 16.07 (6.74) | 6 - 40 |
| Attachment avoidance | 13.75 (6.44) | 6 - 41 |
| Loneliness | 5.19 (2.20) | 3 - 15 |
| Disruptions | 0.65 (0.89) | 0 - 5 |
| Trust | 24.97 (4.98) | 5 - 33 |

**Table S2.**

*Phenotypic Correlations for Social Factors*.

|  | Relationship satisfaction | Loneliness | Attachment anxiety | Attachment avoidance | Disruptions | Trust |
| --- | --- | --- | --- | --- | --- | --- |
| Relationship satisfaction | 1.000 | -.400 | -.339 | -.617 | -.276 | .176 |
| Loneliness | -.400 | 1.000 | .397 | .416 | .238 | -.277 |
| Attachment anxiety | -.339 | .397 | 1.000 | .484 | .199 | -.227 |
| Attachment avoidance | -.617 | .416 | .484 | 1.000 | .169 | -.195 |
| Disruptions | -.276 | .238 | .199 | .169 | 1.000 | -.131 |
| Trust | .176 | -.277 | -.227 | -.195 | -.131 | 1.000 |

**Table S3.**

*Correlations for Monozygotic Twins*.

| **Social factor** | **Twin 1** | **Twin 2** |
| --- | --- | --- |
| Relationship satisfaction | 1.00 | 0.392 |
| Attachment anxiety | 1.00 | 0.359 |
| Attachment avoidance | 1.00 | 0.334 |
| Loneliness | 1.00 | 0.384 |
| Disruptions | 1.00 | 0.242 |
| Trust | 1.00 | 0.332 |

**Table S4.**

*Correlations for Dizygotic Twins*.

| **Social factor** | **Twin 1** | **Twin 2** |
| --- | --- | --- |
| Relationship satisfaction | 1.00 | 0.102 |
| Attachment anxiety | 1.00 | 0.217 |
| Attachment avoidance | 1.00 | 0.221 |
| Loneliness | 1.00 | 0.175 |
| Disruptions | 1.00 | 0.157 |
| Trust | 1.00 | 0.206 |

**Table S5.**

*Fit Statistics for Multivariate Cholesky Models*.

| **Model** | **df** | **p** | **AIC** |
| --- | --- | --- | --- |
| ACE | 69 |  | 27812.99 |
| ADE | 69 |  | 27816.49 |
| AE | 48 | 0.995 | 27779.04 |
| CE | 48 | 0.065 | 27802.53 |
| E | 27 | < 0.001 | 27982.38 |

**Table S6.**

*Parameter Estimates from AE Cholesky model*.

| **Social factor** | **A1** | **A2** | **A3** | **A4** | **A5** | **A6** |
| --- | --- | --- | --- | --- | --- | --- |
| Relationship satisfaction | .577 |  |  |  |  |  |
| Attachment anxiety | -0.255 | 0.551 |  |  |  |  |
| Attachment avoidance | -0.424 | 0.182 | 0.387 |  |  |  |
| Loneliness | -0.407 | 0.220 | 0.008 | 0.449 |  |  |
| Disruptions | -0.417 | -0.065 | -0.122 | 0.004 | 0.224 |  |
| Trust | 0.208 | -0.179 | -0.198 | -0.172 | -0.243 | 0.420 |

**Table S7.**

*Parameter Estimates from AE Cholesky model*.

| **Social factor** | **E1** | **E2** | **E3** | **E4** | **E5** | **E6** |
| --- | --- | --- | --- | --- | --- | --- |
| Relationship satisfaction | 0.817 |  |  |  |  |  |
| Attachment anxiety | -0.255 | 0.753 |  |  |  |  |
| Attachment avoidance | -0.470 | 0.206 | 0.611 |  |  |  |
| Loneliness | -0.238 | 0.156 | 0.116 | 0.700 |  |  |
| Disruptions | -0.084 | 0.149 | -0.006 | 0.061 | 0.851 |  |
| Trust | 0.095 | -0.076 | 0.084 | -0.079 | -0.008 | 0.770 |

**Table S8.**

*Genetic Correlations Estimated in the AE Cholesky model*.

| **Social factor** | **rA1** | **rA2** | **rA3** | **rA4** | **rA5** | **rA6** |
| --- | --- | --- | --- | --- | --- | --- |
| Relationship satisfaction | 1.000 |  |  |  |  |  |
| Attachment anxiety | -0.420 | 1.000 |  |  |  |  |
| Attachment avoidance | -0.705 | 0.570 | 1.000 |  |  |  |
| Loneliness | -0.631 | 0.575 | 0.556 | 1.000 |  |  |
| Disruptions | -0.846 | 0.236 | 0.398 | 0.491 | 1.000 |  |
| Trust | 0.337 | -0.406 | -0.532 | -0.510 | -0.349 | 1.000 |

**Table S9.**

*Environmental Correlations Estimated in the AE Cholesky model*.

| **Social factor** | **rE1** | **rE2** | **rE3** | **rE4** | **rE5** | **rE6** |
| --- | --- | --- | --- | --- | --- | --- |
| Relationship satisfaction | 1.000 |  |  |  |  |  |
| Attachment anxiety | -0.321 | 1.000 |  |  |  |  |
| Attachment avoidance | -0.589 | 0.433 | 1.000 |  |  |  |
| Loneliness | -0.311 | 0.294 | 0.352 | 1.000 |  |  |
| Disruptions | -0.097 | 0.194 | 0.096 | 0.128 | 1.000 |  |
| Trust | 0.121 | -0.130 | -0.014 | -0.133 | -0.045 | 1.000 |

**Table S10.**

*Estimates From Multilevel Model: Relationship Satisfaction (Full Sample) and SWLS 2016*.

| Random effects |  |  |  |  |  |
| --- | --- | --- | --- | --- | --- |
| Groups | Name | Variance | SD |  |  |
| PairID | (Intercept) | 0.0922 | 0.3037 |  |  |
| Residual |  | 0.5591 | 0.7477 |  |  |
| Number of obs: | 1509 |  |  |  |  |
|  |  |  |  |  |  |
| Fixed effects |  |  |  |  |  |
|  | Estimate | SE | df | t-value | p-value |
| Intercept | 0.0276 | 0.3160 | 956.8 | 0.087 | 0.930 |
| scale(RS16) | 0.4661 | 0.0209 | 1493 | 22.273 | 0.000 |
| Sex | -0.0525 | 0.0435 | 960.4 | -1.209 | 0.227 |
| Age (2016) | -0.0028 | 0.0048 | 942.5 | 0.592 | 0.554 |

**Table S11.**

*Estimates From Multilevel Model: Relationship Satisfaction (MZ Twins) and SWLS 2016*.

| Random effects |  |  |  |  |  |
| --- | --- | --- | --- | --- | --- |
| Groups | Name | Variance | SD |  |  |
| PairID | (Intercept) | 0.1280 | 0.3578 |  |  |
| Residual |  | 0.6178 | 0.7860 |  |  |
| Number of obs: | 438 |  |  |  |  |
|  |  |  |  |  |  |
| Fixed effects |  |  |  |  |  |
|  | Estimate | SE | df | t-value | p-value |
| Intercept | 0.9243 | 0.6410 | 215 | 1.442 | 0.1510 |
| scale(RS16-RS16_mean) | 0.2731 | 0.0376 | 218 | 7.262 | 0.0000 |
| scale(RS16_mean) | 0.4250 | 0.0448 | 215 | 9.488 | 0.0000 |
| Sex | -0.1414 | 0.0915 | 215 | -1.546 | 0.1240 |
| Age (2016) | -0.0111 | 0.0095 | 215 | -1.172 | 0.2420 |

**Table S12.**

*Estimates From Multilevel Model: Relationship Satisfaction (Full Sample) and SWLS 2022*.

| Random effects |  |  |  |  |  |
| --- | --- | --- | --- | --- | --- |
| Groups | Name | Variance | SD |  |  |
| PairID | (Intercept) | 0.0640 | 0.2529 |  |  |
| Residual |  | 0.6713 | 0.8193 |  |  |
| Number of obs: | 978 |  |  |  |  |
|  |  |  |  |  |  |
| Fixed effects |  |  |  |  |  |
|  | Estimate | SE | df | t-value | p-value |
| Intercept | 1.0271 | 0.4166 | 668.7 | 2.466 | 0.0139 |
| scale(RS16) | 0.4074 | 0.0278 | 964.7 | 14.630 | 0.0000 |
| Sex | -0.1223 | 0.0565 | 687.4 | -2.165 | 0.0308 |
| Age (2016) | -0.0121 | 0.0063 | 656.4 | -1.911 | 0.0565 |

**Table S13.**

*Estimates From Multilevel Model: Relationship Satisfaction (MZ Twins) and SWLS 2022*.

| Random effects |  |  |  |  |  |
| --- | --- | --- | --- | --- | --- |
| Groups | Name | Variance | SD |  |  |
| PairID | (Intercept) | 0.0349 | 0.1867 |  |  |
| Residual |  | 0.6938 | 0.8329 |  |  |
| Number of obs: | 244 |  |  |  |  |
|  |  |  |  |  |  |
| Fixed effects |  |  |  |  |  |
|  | Estimate | SE | df | t-value | p-value |
| Intercept | 2.5842 | 0.7792 | 118 | 3.317 | 0.0012 |
| scale(RS16-RS16_mean) | 0.1364 | 0.0534 | 121 | 2.552 | 0.0120 |
| scale(RS16_mean) | 0.4779 | 0.0561 | 118 | 8.526 | 0.0000 |
| Sex | -0.1786 | 0.1145 | 118 | -1.560 | 0.1214 |
| Age (2016) | -0.0369 | 0.0117 | 118 | -3.159 | 0.0020 |

**Table S14.**

*Estimates From Multilevel Model: Loneliness (Full Sample) and SWLS 2016*.

| Random effects |  |  |  |  |  |
| --- | --- | --- | --- | --- | --- |
| Groups | Name | Variance | SD |  |  |
| PairID | (Intercept) | 0.1470 | 0.3834 |  |  |
| Residual |  | 0.6871 | 0.8289 |  |  |
| Number of obs: | 1860 |  |  |  |  |
|  |  |  |  |  |  |
| Fixed effects |  |  |  |  |  |
|  | Estimate | SE | df | t-value | p-value |
| Intercept | 0.0271 | 0.3295 | 1108 | 0.082 | 0.9345 |
| scale(lon) | -0.4064 | 0.0214 | 1833 | -18.979 | 0.0000 |
| Sex | -0.0808 | 0.0453 | 1098 | -1.784 | 0.0746 |
| Age (2016) | 0.0017 | 0.0050 | 1089 | 0.336 | 0.7368 |

**Table S15.**

*Estimates From Multilevel Model: Loneliness (MZ Twins) and SWLS 2016*.

| Random effects |  |  |  |  |  |
| --- | --- | --- | --- | --- | --- |
| Groups | Name | Variance | SD |  |  |
| PairID | (Intercept) | 0.1776 | 0.4215 |  |  |
| Residual |  | 0.6629 | 0.8142 |  |  |
| Number of obs: | 630 |  |  |  |  |
|  |  |  |  |  |  |
| Fixed effects |  |  |  |  |  |
|  | Estimate | SE | df | t-value | p-value |
| Intercept | 1.5454 | 0.6030 | 311 | 2.563 | 0.0108 |
| scale(lon-lon_mean) | -0.1975 | 0.0325 | 314 | -6.083 | 0.0000 |
| scale(lon_mean) | -0.3360 | 0.0403 | 311 | -8.327 | 0.0000 |
| Sex | -0.1748 | 0.0838 | 311 | -2.086 | 0.0378 |
| Age (2016) | -0.0200 | 0.0089 | 311 | -2.243 | 0.0256 |

**Table S16.**

*Estimates From Multilevel Model: Loneliness (Full Sample) and SWLS 2022*.

| Random effects |  |  |  |  |  |
| --- | --- | --- | --- | --- | --- |
| Groups | Name | Variance | SD |  |  |
| PairID | (Intercept) | 0.1870 | 0.4324 |  |  |
| Residual |  | 0.6762 | 0.8223 |  |  |
| Number of obs: | 1173 |  |  |  |  |
|  |  |  |  |  |  |
| Fixed effects |  |  |  |  |  |
|  | Estimate | SE | df | t-value | p-value |
| Intercept | 0.8608 | 0.4310 | 836.4 | 1.997 | 0.0461 |
| scale(lon) | -0.3446 | 0.0288 | 1167 | -11.981 | 0.0000 |
| Sex | -0.1274 | 0.0580 | 838.2 | -2.199 | 0.0282 |
| Age (2016) | -0.0105 | 0.0066 | 817.8 | -1.605 | 0.1090 |

**Table S17.**

*Estimates From Multilevel Model: Loneliness (MZ Twins) and SWLS 2022*.

| Random effects |  |  |  |  |  |
| --- | --- | --- | --- | --- | --- |
| Groups | Name | Variance | SD |  |  |
| PairID | (Intercept) | 0.2373 | 0.4872 |  |  |
| Residual |  | 0.6802 | 0.8247 |  |  |
| Number of obs: | 336 |  |  |  |  |
|  |  |  |  |  |  |
| Fixed effects |  |  |  |  |  |
|  | Estimate | SE | df | t-value | p-value |
| Intercept | 2.4231 | 0.8962 | 164 | 2.704 | 0.0076 |
| scale(lon-lon_mean) | -0.0991 | 0.0451 | 167 | -2.199 | 0.0293 |
| scale(lon_mean) | -0.2363 | 0.0592 | 164 | -3.989 | 0.0000 |
| Sex | -0.2540 | 0.1240 | 164 | -2.049 | 0.0421 |
| Age (2016) | -0.0323 | 0.0132 | 164 | -2.438 | 0.0159 |

**Table S18.**

*Estimates From Multilevel Model: Attachment Anxiety (Full Sample) and SWLS 2016*.

| Random effects |  |  |  |  |  |
| --- | --- | --- | --- | --- | --- |
| Groups | Name | Variance | SD |  |  |
| PairID | (Intercept) | 0.0900 | 0.3000 |  |  |
| Residual |  | 0.7574 | 0.8703 |  |  |
| Number of obs: | 1584 |  |  |  |  |
|  |  |  |  |  |  |
| Fixed effects |  |  |  |  |  |
|  | Estimate | SE | df | t-value | p-value |
| Intercept | 0.1079 | 0.3507 | 983.1 | -0.308 | 0.7584 |
| scale(attanx) | -0.3050 | 0.0234 | 1543 | -13.038 | 0.0000 |
| Sex | -0.1403 | 0.0482 | 964.8 | -2.912 | 0.0037 |
| Age (2016) | 0.0060 | 0.0053 | 960.3 | 1.127 | 0.2601 |

**Table S19.**

*Estimates From Multilevel Model: Attachment Anxiety (MZ Twins) and SWLS 2016*.

| Random effects |  |  |  |  |  |
| --- | --- | --- | --- | --- | --- |
| Groups | Name | Variance | SD |  |  |
| PairID | (Intercept) | 0.1113 | 0.3336 |  |  |
| Residual |  | 0.7665 | 0.8755 |  |  |
| Number of obs: | 470 |  |  |  |  |
|  |  |  |  |  |  |
| Fixed effects |  |  |  |  |  |
|  | Estimate | SE | df | t-value | p-value |
| Intercept | 1.3886 | 0.6941 | 231 | 2.000 | 0.0466 |
| scale(attanx-attanx_mean) | -0.1665 | 0.0404 | 234 | -4.118 | 0.0000 |
| scale(attanx_mean) | -0.2884 | 0.0460 | 231 | -6.265 | 0.0000 |
| Sex | -0.2695 | 0.0954 | 231 | -2.825 | 0.0051 |
| Age (2016) | -0.0153 | 0.0102 | 231 | -1.495 | 0.1364 |

**Table S20.**

*Estimates From Multilevel Model: Attachment Anxiety (Full Sample) and SWLS 2022*.

| Random effects |  |  |  |  |  |
| --- | --- | --- | --- | --- | --- |
| Groups | Name | Variance | SD |  |  |
| PairID | (Intercept) | 0.1228 | 0.3505 |  |  |
| Residual |  | 0.7427 | 0.8618 |  |  |
| Number of obs: | 1039 |  |  |  |  |
|  |  |  |  |  |  |
| Fixed effects |  |  |  |  |  |
|  | Estimate | SE | df | t-value | p-value |
| Intercept | 0.5969 | 0.4476 | 769.1 | 1.334 | 0.1828 |
| scale(attanx) | -0.2929 | 0.0296 | 1023 | -9.900 | 0.0000 |
| Sex | -0.1627 | 0.0604 | 753.2 | -2.695 | 0.0072 |
| Age (2016) | -0.0053 | 0.0068 | 746.9 | -0.774 | 0.4393 |

**Table S21.**

*Estimates From Multilevel Model: Attachment Anxiety (MZ Twins) and SWLS 2022*.

| Random effects |  |  |  |  |  |
| --- | --- | --- | --- | --- | --- |
| Groups | Name | Variance | SD |  |  |
| PairID | (Intercept) | 0.1519 | 0.3897 |  |  |
| Residual |  | 0.7588 | 0.8711 |  |  |
| Number of obs: | 266 |  |  |  |  |
|  |  |  |  |  |  |
| Fixed effects |  |  |  |  |  |
|  | Estimate | SE | df | t-value | p-value |
| Intercept | 3.1253 | 0.9768 | 129 | 3.199 | 0.0017 |
| scale(attanx-attanx_mean) | -0.0683 | 0.0535 | 132 | -1.276 | 0.2042 |
| scale(attanx_mean) | -0.2447 | 0.0637 | 129 | -3.842 | 0.0002 |
| Sex | -0.3868 | 0.1333 | 129 | -2.901 | 0.0044 |
| Age (2016) | -0.0403 | 0.0144 | 129 | -2.804 | 0.0058 |

**Table S22.**

*Estimates From Multilevel Model: Attachment Avoidance (Full Sample) and SWLS 2016*.

| Random effects |  |  |  |  |  |
| --- | --- | --- | --- | --- | --- |
| Groups | Name | Variance | SD |  |  |
| PairID | (Intercept) | 0.0829 | 0.2879 |  |  |
| Residual |  | 0.6952 | 0.8338 |  |  |
| Number of obs: | 1671 |  |  |  |  |
|  |  |  |  |  |  |
| Fixed effects |  |  |  |  |  |
|  | Estimate | SE | df | t-value | p-value |
| Intercept | -0.3158 | 0.3293 | 992.5 | -0.959 | 0.3377 |
| scale(attavoi) | -0.3781 | 0.0218 | 1633 | -17.329 | 0.0000 |
| Sex | -0.1237 | 0.0450 | 989.9 | -2.748 | 0.0061 |
| Age (2016) | 0.0091 | 0.0050 | 974.4 | 1.819 | 0.0693 |

**Table S23.**

*Estimates From Multilevel Model: Attachment Avoidance (MZ Twins) and SWLS 2016*.

| Random effects |  |  |  |  |  |
| --- | --- | --- | --- | --- | --- |
| Groups | Name | Variance | SD |  |  |
| PairID | (Intercept) | 0.1007 | 0.3173 |  |  |
| Residual |  | 0.7275 | 0.8529 |  |  |
| Number of obs: | 516 |  |  |  |  |
|  |  |  |  |  |  |
| Fixed effects |  |  |  |  |  |
|  | Estimate | SE | df | t-value | p-value |
| Intercept | 1.0778 | 0.6326 | 254 | 1.704 | 0.0896 |
| scale(attavoi-attavoi_mean) | -0.2212 | 0.0376 | 257 | -5.884 | 0.0000 |
| scale(attavoi_mean) | -0.3381 | 0.0428 | 254 | -7.905 | 0.0000 |
| Sex | -0.2371 | 0.0877 | 254 | -2.704 | 0.0073 |
| Age (2016) | -0.0111 | 0.0094 | 254 | -1.189 | 0.2356 |

**Table S24.**

*Estimates From Multilevel Model: Attachment Avoidance (Full Sample) and SWLS 2022*.

| Random effects |  |  |  |  |  |
| --- | --- | --- | --- | --- | --- |
| Groups | Name | Variance | SD |  |  |
| PairID | (Intercept) | 0.0784 | 0.2800 |  |  |
| Residual |  | 0.7553 | 0.8691 |  |  |
| Number of obs: | 1087 |  |  |  |  |
|  |  |  |  |  |  |
| Fixed effects |  |  |  |  |  |
|  | Estimate | SE | df | t-value | p-value |
| Intercept | 0.5794 | 0.4266 | 767.5 | 1.358 | 0.1748 |
| scale(attavoi) | -0.3264 | 0.0285 | 1050 | -11.470 | 0.0000 |
| Sex | -0.2024 | 0.0574 | 757.1 | -3.525 | 0.0004 |
| Age (2016) | -0.0038 | 0.0065 | 747.6 | -0.579 | 0.5626 |

**Table S25.**

*Estimates From Multilevel Model: Attachment Avoidance (MZ Twins) and SWLS 2022*.

| Random effects |  |  |  |  |  |
| --- | --- | --- | --- | --- | --- |
| Groups | Name | Variance | SD |  |  |
| PairID | (Intercept) | 0.1021 | 0.3196 |  |  |
| Residual |  | 0.7540 | 0.8683 |  |  |
| Number of obs: | 290 |  |  |  |  |
|  |  |  |  |  |  |
| Fixed effects |  |  |  |  |  |
|  | Estimate | SE | df | t-value | p-value |
| Intercept | 2.1820 | 0.8714 | 141 | 2.504 | 0.0134 |
| scale(attavoi-attavoi_mean) | -0.1190 | 0.0511 | 144 | -2.330 | 0.0212 |
| scale(attavoi_mean) | -0.3342 | 0.0580 | 141 | -5.765 | 0.0000 |
| Sex | -0.3168 | 0.1198 | 141 | -2.645 | 0.0091 |
| Age (2016) | -0.0270 | 0.0129 | 141 | -2.088 | 0.0386 |

**Table S26.**

*Estimates From Multilevel Model: Trust (Full Sample) and SWLS 2016*.

| Random effects |  |  |  |  |  |
| --- | --- | --- | --- | --- | --- |
| Groups | Name | Variance | SD |  |  |
| PairID | (Intercept) | 0.1470 | 0.3835 |  |  |
| Residual |  | 0.7826 | 0.8847 |  |  |
| Number of obs: | 1894 |  |  |  |  |
|  |  |  |  |  |  |
| Fixed effects |  |  |  |  |  |
|  | Estimate | SE | df | t-value | p-value |
| Intercept | 0.0355 | 0.3437 | 1149 | -0.103 | 0.9178 |
| scale(trust) | 0.2437 | 0.0225 | 1873 | 10.824 | 0.0000 |
| Sex | -0.1561 | 0.0475 | 1147 | -3.289 | 0.0010 |
| Age (2016) | 0.0044 | 0.0052 | 1127 | 0.848 | 0.3967 |

**Table S27.**

*Estimates From Multilevel Model: Trust (MZ Twins) and SWLS 2016*.

| Random effects |  |  |  |  |  |
| --- | --- | --- | --- | --- | --- |
| Groups | Name | Variance | SD |  |  |
| PairID | (Intercept) | 0.1869 | 0.4323 |  |  |
| Residual |  | 0.7265 | 0.8523 |  |  |
| Number of obs: | 648 |  |  |  |  |
|  |  |  |  |  |  |
| Fixed effects |  |  |  |  |  |
|  | Estimate | SE | df | t-value | p-value |
| Intercept | 1.3452 | 0.6279 | 320 | 2.142 | 0.0329 |
| scale(trust-trust_mean) | 0.0874 | 0.0335 | 323 | 2.608 | 0.0095 |
| scale(trust_mean) | 0.2694 | 0.0415 | 320 | 6.490 | 0.0000 |
| Sex | -0.2988 | 0.0872 | 320 | -3.426 | 0.0007 |
| Age (2016) | -0.0137 | 0.0093 | 320 | -1.473 | 0.1417 |

**Table S28.**

*Estimates From Multilevel Model: Trust (Full Sample) and SWLS 2022*.

| Random effects |  |  |  |  |  |
| --- | --- | --- | --- | --- | --- |
| Groups | Name | Variance | SD |  |  |
| PairID | (Intercept) | 0.1334 | 0.3653 |  |  |
| Residual |  | 0.7951 | 0.8917 |  |  |
| Number of obs: | 1191 |  |  |  |  |
|  |  |  |  |  |  |
| Fixed effects |  |  |  |  |  |
|  | Estimate | SE | df | t-value | p-value |
| Intercept | 0.7103 | 0.4374 | 838.2 | 1.624 | 0.1048 |
| scale(trust) | 0.2539 | 0.0292 | 1184 | 8.691 | 0.0000 |
| Sex | -0.2134 | 0.0592 | 837.3 | -3.607 | 0.0003 |
| Age (2016) | -0.0062 | 0.0066 | 816.8 | -0.932 | 0.3516 |

**Table S29.**

*Estimates From Multilevel Model: Trust (MZ Twins) and SWLS 2022*.

| Random effects |  |  |  |  |  |
| --- | --- | --- | --- | --- | --- |
| Groups | Name | Variance | SD |  |  |
| PairID | (Intercept) | 0.2161 | 0.4649 |  |  |
| Residual |  | 0.7210 | 0.8491 |  |  |
| Number of obs: | 356 |  |  |  |  |
|  |  |  |  |  |  |
| Fixed effects |  |  |  |  |  |
|  | Estimate | SE | df | t-value | p-value |
| Intercept | 2.1999 | 0.8797 | 174 | 2.501 | 0.0133 |
| scale(trust-trust_mean) | 0.0090 | 0.0451 | 176 | 0.199 | 0.8427 |
| scale(trust_mean) | 0.2301 | 0.0577 | 174 | 3.992 | 0.0000 |
| Sex | -0.3456 | 0.1218 | 174 | -2.838 | 0.0051 |
| Age (2016) | -0.0263 | 0.0129 | 174 | -2.036 | 0.0433 |

**Table S30.**

*Estimates From Multilevel Model: Disruption Last Year (Full Sample) and SWLS 2016*.

| Random effects |  |  |  |  |  |
| --- | --- | --- | --- | --- | --- |
| Groups | Name | Variance | SD |  |  |
| PairID | (Intercept) | 0.1955 | 0.4421 |  |  |
| Residual |  | 0.7287 | 0.8536 |  |  |
| Number of obs: | 1843 |  |  |  |  |
|  |  |  |  |  |  |
| Fixed effects |  |  |  |  |  |
|  | Estimate | SE | df | t-value | p-value |
| Intercept | 0.2742 | 0.3532 | 1172 | 0.776 | 0.4377 |
| disrupt_lastyr | -0.6569 | 0.0754 | 1836 | -8.717 | 0.0000 |
| Sex | -0.0964 | 0.0483 | 1157 | -1.997 | 0.0461 |
| Age (2016) | -0.0006 | 0.0054 | 1150 | -0.108 | 0.9138 |

**Table S31.**

*Estimates From Multilevel Model: Disruption Last Year (MZ Twins) and SWLS 2016*.

| Random effects |  |  |  |  |  |
| --- | --- | --- | --- | --- | --- |
| Groups | Name | Variance | SD |  |  |
| PairID | (Intercept) | 0.2657 | 0.5155 |  |  |
| Residual |  | 0.6803 | 0.8248 |  |  |
| Number of obs: | 616 |  |  |  |  |
|  |  |  |  |  |  |
| Fixed effects |  |  |  |  |  |
|  | Estimate | SE | df | t-value | p-value |
| Intercept | 1.5205 | 0.6785 | 304 | 2.241 | 0.0258 |
| (disrupt_lastyr-disrupt_lastyr_mean) | -0.5290 | 0.1800 | 307 | -2.939 | 0.0035 |
| (disrupt_lastyr_mean) | -0.3769 | 0.2677 | 604.8 | -1.408 | 0.1597 |
| Sex | -0.1909 | 0.0924 | 304 | -2.065 | 0.0397 |
| Age (2016) | -0.0179 | 0.0100 | 304 | -1.782 | 0.0758 |

**Table S32.**

*Estimates From Multilevel Model: Disruption Last Year (Full Sample) and SWLS 2022*.

| Random effects |  |  |  |  |  |
| --- | --- | --- | --- | --- | --- |
| Groups | Name | Variance | SD |  |  |
| PairID | (Intercept) | 0.1775 | 0.4214 |  |  |
| Residual |  | 0.7715 | 0.8783 |  |  |
| Number of obs: | 1155 |  |  |  |  |
|  |  |  |  |  |  |
| Fixed effects |  |  |  |  |  |
|  | Estimate | SE | df | t-value | p-value |
| Intercept | 0.9312 | 0.4533 | 818.9 | 2.054 | 0.0403 |
| (disrupt_lastyr) | -0.5754 | 0.0981 | 1145 | -5.867 | 0.0000 |
| Sex | -0.1668 | 0.0608 | 815.5 | -2.745 | 0.0062 |
| Age (2016) | -0.0096 | 0.0069 | 800.2 | -1.386 | 0.1663 |

**Table S33.**

*Estimates From Multilevel Model: Disruption Last Year (MZ Twins) and SWLS 2022*.

| Random effects |  |  |  |  |  |
| --- | --- | --- | --- | --- | --- |
| Groups | Name | Variance | SD |  |  |
| PairID | (Intercept) | 0.2516 | 0.5016 |  |  |
| Residual |  | 0.6964 | 0.8345 |  |  |
| Number of obs: | 326 |  |  |  |  |
|  |  |  |  |  |  |
| Fixed effects |  |  |  |  |  |
|  | Estimate | SE | df | t-value | p-value |
| Intercept | 2.5504 | 0.9348 | 159 | 2.728 | 0.0071 |
| (disrupt_lastyr-disrupt_lastyr_mean) | -0.1561 | 0.2461 | 162 | -0.634 | 0.5269 |
| (disrupt_lastyr_mean) | -0.7755 | 0.3785 | 312.6 | -2.049 | 0.0413 |
| Sex | -0.2361 | 0.1269 | 159 | -1.860 | 0.0647 |
| Age (2016) | -0.0334 | 0.0138 | 159 | -2.414 | 0.0169 |

**Table S34.**

*Estimates From Multilevel Model: Disruption Previously (Full Sample)* *and SWLS 2016*.

| Random effects |  |  |  |  |  |
| --- | --- | --- | --- | --- | --- |
| Groups | Name | Variance | SD |  |  |
| PairID | (Intercept) | 0.1595 | 0.3993 |  |  |
| Residual |  | 0.7965 | 0.8925 |  |  |
| Number of obs: | 1899 |  |  |  |  |
|  |  |  |  |  |  |
| Fixed effects |  |  |  |  |  |
|  | Estimate | SE | df | t-value | p-value |
| Intercept | 0.1886 | 0.3509 | 1178 | 0.537 | 0.5911 |
| (disrupt_prev) | -0.3452 | 0.0465 | 1893 | -7.423 | 0.0000 |
| Sex | -0.0959 | 0.0479 | 1165 | -2.000 | 0.0457 |
| Age (2016) | 0.0015 | 0.0053 | 1150 | 0.282 | 0.7781 |

**Table S35.**

*Estimates From Multilevel Model: Disruption Previously (MZ Twins) and SWLS 2016*.

| Random effects |  |  |  |  |  |
| --- | --- | --- | --- | --- | --- |
| Groups | Name | Variance | SD |  |  |
| PairID | (Intercept) | 0.2056 | 0.4535 |  |  |
| Residual |  | 0.7303 | 0.8546 |  |  |
| Number of obs: | 656 |  |  |  |  |
|  |  |  |  |  |  |
| Fixed effects |  |  |  |  |  |
|  | Estimate | SE | df | t-value | p-value |
| Intercept | 1.7540 | 0.6408 | 324 | 2.737 | 0.0065 |
| (disrupt_prev-disrupt_prev_mean) | -0.0103 | 0.1081 | 327 | -0.095 | 0.9244 |
| (disrupt_prev_mean) | -0.6475 | 0.1568 | 649.1 | -4.129 | 0.0000 |
| Sex | -0.2228 | 0.0874 | 324 | -2.548 | 0.0113 |
| Age (2016) | -0.0183 | 0.0094 | 324 | -1.947 | 0.0524 |

**Table S36.**

*Estimates From Multilevel Model: Disruption Previously (Full Sample)* *and SWLS 2022*.

| Random effects |  |  |  |  |  |
| --- | --- | --- | --- | --- | --- |
| Groups | Name | Variance | SD |  |  |
| PairID | (Intercept) | 0.1700 | 0.4123 |  |  |
| Residual |  | 0.7819 | 0.8842 |  |  |
| Number of obs: | 1190 |  |  |  |  |
|  |  |  |  |  |  |
| Fixed effects |  |  |  |  |  |
|  | Estimate | SE | df | t-value | p-value |
| Intercept | 0.9122 | 0.4493 | 851.3 | 2.030 | 0.0426 |
| (disrupt_prev) | -0.3632 | 0.0587 | 1180 | -6.186 | 0.0000 |
| Sex | -0.1377 | 0.0601 | 844.8 | -2.290 | 0.0223 |
| Age (2016) | -0.0090 | 0.0068 | 828.5 | -1.317 | 0.1881 |

**Table S37.**

*Estimates From Multilevel Model: Disruption Previously (MZ Twins) and SWLS 2022*.

| Random effects |  |  |  |  |  |
| --- | --- | --- | --- | --- | --- |
| Groups | Name | Variance | SD |  |  |
| PairID | (Intercept) | 0.2349 | 0.4846 |  |  |
| Residual |  | 0.7185 | 0.8476 |  |  |
| Number of obs: | 352 |  |  |  |  |
|  |  |  |  |  |  |
| Fixed effects |  |  |  |  |  |
|  | Estimate | SE | df | t-value | p-value |
| Intercept | 2.6449 | 0.9214 | 172 | 2.870 | 0.0046 |
| (disrupt_prev-disrupt_prev_mean) | -0.0643 | 0.1403 | 175 | -0.458 | 0.6472 |
| (disrupt_prev_mean) | -0.4596 | 0.2147 | 338.9 | -2.141 | 0.0330 |
| Sex | -0.2657 | 0.1231 | 172 | -2.158 | 0.0323 |
| Age (2016) | -0.0324 | 0.0135 | 172 | -2.404 | 0.0173 |
